# Effect of inoculation with a microbial consortium that degrades organic acids on the composting efficiency of food waste

**DOI:** 10.1111/1751-7915.13294

**Published:** 2018-07-02

**Authors:** Caihong Song, Yali Zhang, Xunfeng Xia, Hui Qi, Mingxiao Li, Hongwei Pan, Beidou Xi

**Affiliations:** ^1^ Life Science College Liaocheng University No. 1, Hunan Road, Dongchangfu District Liaocheng China; ^2^ Institute of Geographic Sciences and Natural Resources Research Chinese Academy of Sciences Beijing 100101 China; ^3^ Center for Chinese Agricultural Policy Chinese Academy of Sciences Beijing 100101 China; ^4^ Innovation Base of Groundwater and Environmental Systems Engineering Chinese Research Academy of Environmental Sciences No. 8, Dayangfang, Beiyuan Road, Chaoyang District Beijing 100012 China; ^5^ North China University of Water Resources and Electric Power Zhengzhou 450011 China

## Abstract

In order to overcome the excessive acidification problem, a microbial consortium for the degradation of organic acids (MCDOA), which acts synergistically in degrading organic acids, was developed and used as an inoculum to improve the efficiency of food waste composting. MCDOA could eliminate the initial lag phase of the pile temperature rise because of excessive acidification and effectively shorten the composting period. Fluorescence regional integration analysis of the excitation‐emission matrix spectra of dissolved organic matter showed that compared with raw material, in compost with MCDOA inoculation, the percent fluorescence response (*P*_i,n_) values of Regions I, II and IV decreased by 95.11%, 94.19% and 87.41%, respectively, and *P*_i,n_ of Region V increased by 172.57%. The decreased and increased levels were markedly higher than in the two control groups (MgO and K_2_ HPO_4_ treatment, and uninoculated compost). These findings revealed that MCDOA accelerated the degradation of proteinaceous compounds and the formation of complicated humic‐like materials. Bacterial profiles implied that MCDOA could improve the indigenous bacterial community structure and diversities of acetic and propionic acid‐degrading and lignin‐degrading bacteria, which might account for the high composting efficiency and degree of humification of the inoculated compost.

## Introduction

In recent years, with the rapid development of the economy and the increase in population, a large amount of food waste has been generated from households, restaurants, markets and many other food processing factories in China (Wei *et al*., 2016a; Wang *et al*., 2018). Food waste contains a high proportion of biodegradable organic compounds and can cause hygienic hazards, odor and ground water pollution from the leaching of pollutants if not properly treated. Composting is viewed as a clean and viable method to circulate organic wastes (Zhang *et al*., 2016; Wu *et al*., 2017) and is often used to dispose of food waste and achieve nutrient recycling.

One of the significant characteristics of food waste composting is a decrease in pH in the early stages of composting (Sundberg *et al*., 2013; Wang *et al*., 2016). The reasons for this decrease in pH are as follows: (i) food waste contains abundant and easily degradable substances, such as sugars, fats, starches and grease. Degradation of these substances produces a large amount of acidic intermediate by‐products, termed organic acids, which lead to the reduced pH of the composting substrate at the initial stage of food waste composting, (ii) Some bacterial species, such as *Escherichia coli* and *Lactobacillus*, are capable of oxidizing organic matter to produce short‐chain organic acids under aerobic conditions and (iii) Since anaerobic microenvironments often exist in the aerobic composting matrix due to the restriction of oxygen flow, organic acids can be produced by obligate or facultative anaerobes (Cheung *et al*., 2010). The presence of organic acids and low pH values could lead to the retarded decomposition rate of organic matter and thereby lower the composting efficiency due to reduced microbial activities.

In order to cope with the problems caused by low pH during food waste composting, several authors have suggested that chemical substances, such as MgO and K_2_HPO_4_ (Wang *et al*., 2013), coal ash and uric acid (An *et al*., 2012) and Ca(OH)_2_ (Bergersen *et al*., 2009), could be added to prevent the decrease in pH. However, the addition of chemical substances easily leads to a high electrical conductivity of the compost, so this must be further investigated to improve the approach. Aasen *et al*. (2006) reported that increased porosity in the composting mix also favored the consumption of short‐chain organic acids by microorganisms, and thereby alleviated the drop in pH. However, the addition of extra bulking agents might bring about some problems, such as the demand of a larger stock and the additional cost of purchasing bulking agent for large‐scale composting plants.

Inoculating pure cultured microbial strains is also deemed an alternative approach to prevent the drop in pH, and thereby the efficiency of food waste composting. Choi and Park (1998) showed that *Kluyveromyces marxianus* reduced the acidity of food waste compost and increased the quantity of indigenous thermophilic bacteria in the composting substrate. Nevertheless, they were unable to determine whether *Kluyveromyces marxianus* influenced the thermophilic bacteria, which become dominant in the thermophilic phase of composting. This was due to a lack of techniques to enable the comparison of microbial population structures. Nakasaki *et al*. (2013) showed that short‐chain organic acids could be rapidly degraded by the inoculation of *Pichia kudriavzevii* RB1.The initial lag phase seen in the growth of indigenous microorganisms was also eliminated, and the composting period was reduced by 2 days. However, because high temperature was lethal for the yeast, it died in the early stages of composting and did not contribute during the thermophilic phase of composting. In order to avoid the accumulation of organic acids after the death of the yeast, Nakasaki and Hirai (2017) tried to prevent the death of the inoculated yeast by maintaining the temperature at 40°C for 2 days during the heating stage in the initial phase of composting and also showed that controlling the composting temperature, in addition to inoculating the yeast, was effective at accelerating the composting process. As was well known, the production of organic acids depended strongly on the amount of easily degradable carbonaceous materials in the raw compost materials. Thus, the time during which the temperature was maintained at mesophilic temperature depended on the composition of the raw compost material. Additionally, the lactic acid bacterium *Pediococcus acidilactici* TM14 could avoid the accumulation of organic acids in an indirect way (Tran *et al*., 2015). That is, it stimulated the QH1 (a fungus that was indigenous to the raw compost material), which in turn degraded all the organic acids and modified the pH and the environmental conditions under which the other microorganisms operated. The magnitude of the effect of the lactic acid bacterium on the indigenous microorganisms may be dependent on the raw composting materials.

In this study, in order to improve the survival rate of the inoculated microbes in the course of competing with indigenous microbes for nutrients and further to accelerate the degradation of organic matter, a microbial consortium was developed, which acts synergistically for the rapid bioconversion of organic acids. It originated from the initial phase of food waste composting when the pH of composting material was maintained in the range of 4–5 for a long period of time. Theoretically, the microbial consortium was well adapted to the food‐waste composting environment and vigorously degraded organic acids. In the present study, the microbial consortium was used as an inoculum to eliminate the negative influence of the accumulation of organic acids during the degradation of organic matter in the course of food waste composting. Meanwhile, composting efficiency and the degree of humification of the compost were compared with uninoculated composting treatments. In addition, bacterial succession over time was studied by the polymerase chain reaction‐denaturing gradient gel electrophoresis (PCR–DGGE) method. The objectives of this study were twofold: (i) to evaluate the effect of inoculation with the microbial consortium that degrades organic acids (MCDOA) on the improvement of food waste composting efficiency and the degree of compost humification and (ii) to explore the mechanism by which the inoculation of MCDOA in food waste composting impacts the rate of degradation of organic matter. To the best of our knowledge, this is the first study demonstrating the effects of the inoculation of a microbial consortium that has a high degradation activity against organic acids in food waste composting, specifically with respect to the quantitative analysis of the degradation of organic matter and the formation of humic substances, and the microbial community structure.

## Results and discussion

### Changes in composting temperature

**Figure 1:**
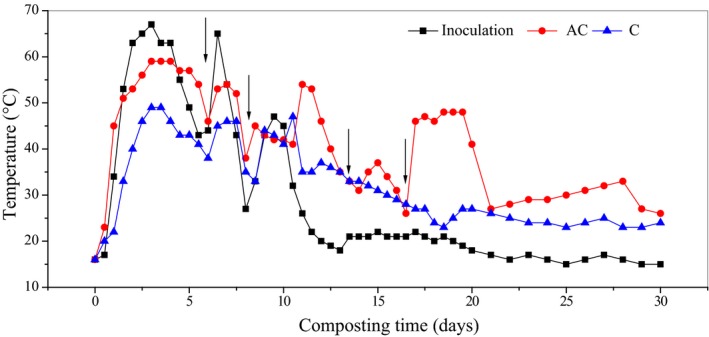
The changes in temperature during the food waste composting process, Inoculation – the inoculation with MCDOA, AC – the alkaline compound treatment, C–the control group. The arrows represent the turning.

The temperature change in a composting pile is closely correlated to the microbial activities and is normally considered to be one of the main parameters used to monitor the composting efficiency (Bustamante *et al*., 2008). Within a certain range, for every 10°C increase in temperature, the microbial metabolic rate will be increased by time (López‐González *et al*., 2013). The temperature changes in the course of composting are shown in Fig. 1. The main concern of this study was the initial lag phase of the rise in the pile temperature. Figure 1 only shows the temperature changes during a period of 30 days. After a 30‐day composting period, the pile temperatures were all below 30°C in three composting treatments (data not shown).

In the inoculation group (Inoculation), the pile temperature reached the thermophilic stage (exceeding 50°C) after only 32 h of composting. The lag phase of pile temperature rise, which often has been reported in food waste composting (Nakasaki *et al*., 2013; Wang *et al*., 2016), was not observed. The disruption of the temperature increase was caused by the inhibition of the mesophiles while the thermophiles had not sufficiently developed (Tran *et al*., 2015). This indicated that inhibition of the activity of the mesophiles did not occur in the Inoculation group. In the alkaline compound treatment group (AC), although the highest temperature (59°C) of the composting pile was lower than that of the Inoculation group (67°C), the composting temperature could reach the thermophilic stage after 32 h of composting and the disruption of pile temperature increase could also be avoided. However, in the control group (C), the pile temperature was lower than 50°C during the entire composting process. According to the US EPA (USEPA, 1994), the Inoculation and AC treatment could meet hygienic requirements for organic waste on days 5 and 6, respectively, whereas the C group could not.

### Changes in chemical and humification parameters

#### pH

**Figure 2:**
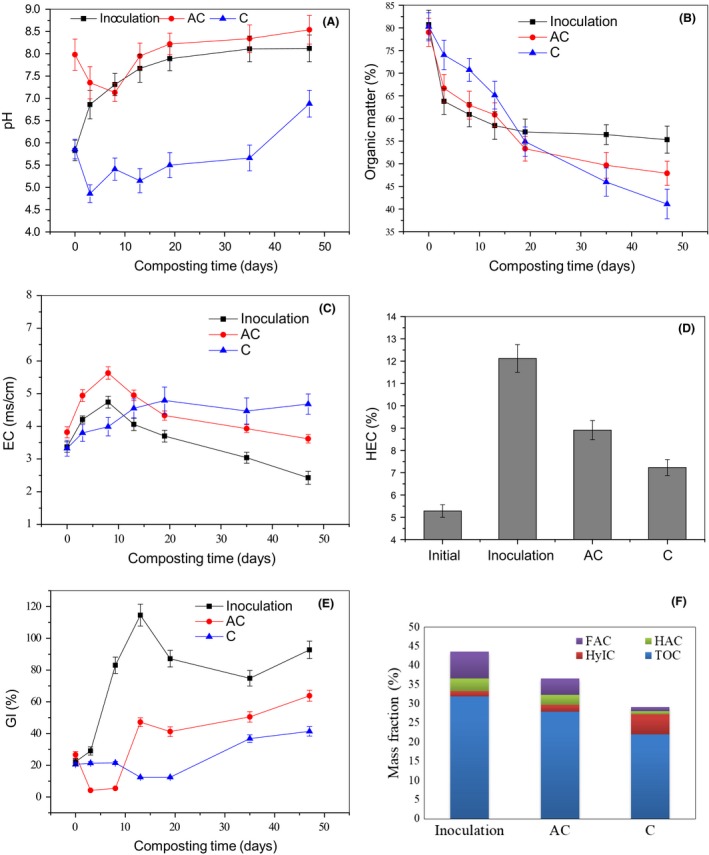
Comparison of the variation in pH (A), OM (B), EC (C), HEC (D) and GI (E) during different composting treatments. (F) The composition of carbon fractions of compost originated from different composting treatments. Inoculation–the inoculation with MCDOA, AC – the alkaline compound treatment, C – the control group, OM – organic matter; EC – electrical conductivity; HEC – humic‐like extract carbon; GI – Germination index; TOC – total organic carbon; HyIC – hydrophilic (HyI) fraction carbon; HAC – humic‐like acid carbon; FAC – fulvic‐like acid carbon. The data represent the mean of three replicates and the error bars are the standard deviation.

The pH range that was suggested as suitable for composting was 6–8 (Bustamante *et al*., 2008). Changes in pH during different composting treatments are presented in Fig. 2A. The pH in the Inoculation group increased continuously after composting started and finally reached a steady state (around 8.1) after day 35. In general, an obvious pH drop would be observed in the initial stage of composting, especially in food waste composting, because low molecular weight organic acids were substantially produced (Cheung *et al*., 2010). In this study, once produced, the low molecular weight organic acids could be rapidly degraded by the microorganisms (Table S1). Thus, accumulation of organic acids and the pH drop did not occur in the Inoculation treatment. In the AC treatment, because of the addition of alkaline compounds, the initial pH (7.98) was markedly higher than the Inoculation (5.83) and C (5.86) groups. During the initial 8 days of composting, a pH decrease (on day 8, the pH decreased to 7.13) was observed in the AC treatment due to the production of a large amount of low molecular weight organic acids. Thereafter, as low molecular weight organic acids were gradually exhausted (Table S1), the pH of the compost increased until composting ended and was higher than the Inoculation group on day 13. By coupling the temperature profile, it could be inferred that alkaline materials provided better pH buffering as additives in the AC group. In the C group, an obvious acidification process was observed (Table S1). On day 3, the pH decreased to the lowest value (4.86). Thereafter, although it displayed an upward trend, the pH was acidic during the entire composting process.

#### Organic matter

To a certain extent, the dynamic changes in the organic matter indicated the composting process (Zhao *et al*., 2016). At the same time, the organic matter status of the compost also had an important significance for the physical and chemical properties of the soil. Changes in the organic matter during different composting treatments are presented in Fig. 2B. During the entire composting process, the organic matter decreased in all three treatments. The loss of organic matter obeyed the following order: Inoculation (24.59%) > AC (20.34%) > C (11.91%) after day 8. The C group had the lowest loss of organic matter. It is well known that low pH inhibits the activities of the microorganisms that are the main contributors to the degradation of organic material during composting (Tran *et al*., 2015). This may be responsible for the low degradation rate of organic matter and composting efficiency of the C group in the early stage of composting. In the Inoculation group, because of the absence of accumulation of organic acids and the pH drop (see the ‘pH’ section), the problem of inhibition of microbial activity did not appear, and this might account for its high loss of organic matter. The AC group exhibited a high pH (> 7.13) during the entire course of composting, which also may be responsible for its higher loss of organic matter. Different from the early stage, from day 13 to the end of composting the loss of organic matter in the Inoculation group (5.34%) was significantly lower than in the AC (21.25%) and C (36.89%) groups. At the end of composting, the organic matter content obeyed the following order: Inoculation (up to 55.32%) > AC (47.90%) > C (41.44%). Xi *et al*. (2012) suggested that as easily degradable organic matter was consumed promptly by active microbial metabolism in the early stage of composting, the composting substrate primarily contained refractory organic matter, such as cellulose and lignin, in the later stage and the degradation of organic matter slowed down. At the same time, the formation of humic substances became vigorous (Gou *et al*., 2017). The aforementioned results suggest that the inoculation of MCDOA may be beneficial to both the degradation of organic matter in the initial phase and the formation of humic substances at the late stage of food waste composting (this idea will be demonstrated in the sections titled ‘Humic‐like extract carbon (HEC)’ and ‘The composition of carbon fractions’). C/N is an important indicator parameter for the biodegradation process and composting efficiency. According to Fig. S1, at the end of composting, the C/N of the Inoculation compost was 13.52, markedly lower than AC (16.03) and C compost (19.37), which implied a high composting efficiency and an accelerated composting process in the Inoculation group. Alkaline materials as additives could provide pH buffering and more favorable conditions for the degradation of easily degradable substances and humification of the composting substrates to a certain extent. In the C group, as the pH increased slowly (as shown in Fig. 2A), the degradation of organic matter became more active in the late stage of composting. However, the C group exhibited low composting efficiency.

#### Electrical conductivity (EC)

Compost that had high EC would detrimentally influence soil microorganisms. Changes in EC during different composting treatments are presented in Fig. 2C. Compost EC increased first and then decreased in both the inoculation and AC groups. This was consistent with the results of Hosseini and Aziz (2013). In the early stage of composting, as easily degradable substances such as aliphatics, proteins and polysaccharides were degraded, the EC increased rapidly. Thereafter, because small molecular substances were exhausted, the EC started to decline gradually. In the AC group, EC was markedly higher than in the Inoculation treatment during the entire composting process, which was attributed to the addition of alkaline compounds. In the C group, EC increased continuously during composting. Compared with the other two treatments, it was markedly lower during the initial 8 days of composting and higher from day 19 to day 47. As revealed in the section ‘Organic matter’, these results might indicate a slow composting process and low composting efficiency in the C group. Gao *et al*. (2010) reported that when the compost EC was < 3.0 mS/cm, plants could grow best. At the end of composting, the EC was in the order Inoculation <AC <C. Except for the Inoculation group, the EC was higher than 3.0 mS/cm in the other two treatments.

#### Humic‐like extract carbon (HEC)

As shown in Fig. 2D, the HEC of the three groups increased significantly after composting. Compared with the C group, HEC increased by 67.63% in the Inoculation treatment, which indicated that the inoculation of MCDOA could significantly increase the HEC content of compost. This finding corroborated the aforementioned inference (in the section ‘Organic matter’) that the MCDOA inoculum could accelerate the formation of humic substances. Compared with the C group, the HEC increased by 23.24% in the AC treatment. This implied that the addition of alkaline compounds could also promote the synthesis of humic substances in compost to a certain extent.

#### Germination index (GI)

The GI is a sensitive indicator reflecting the phytotoxicity of compost (Song *et al*., 2015). As shown in Fig. 2E, on day 8, the GI was 83.01% in the Inoculation group. However, it was < 10% in the AC treatment. On day 13, the GI of the inoculation and AC groups was up to 111% and about 40% respectively. According to the conclusion originating from the ‘Changes in composting temperature’ section, the Inoculation treatment could meet the hygienic requirements for organic waste on day 5. A GI value higher than 50% has been used as an indicator of mature compost (Zucconi *et al*., 1981). According to this standard, it could be considered that the Inoculation compost was mature on day 8. These findings implied that the inoculation of MCDOA could significantly enhance the efficiency of food waste composting and effectively shorten the composting period. Although the GI of the AC and C groups showed an upward trend in general during composting, it reached 50.50% in the AC treatment on day 35 and did not reach 50% in the C group by day 47.

#### The composition of carbon fractions

The composition of organic matter can reflect the quality of compost and is of great significance in the land utilization of compost. Figure 2E shows the distribution of the carbon fractions of three composts. The total organic carbon (TOC) of the three composts was in the order Inoculation (31.90%) > AC (27.88%) > C (21.98%), which was consistent with the result of organic matter at the end of composting. Hydrophilic fraction carbon (HyIC) with a low degree of humification was markedly lower in the Inoculation (1.42%) and AC (1.85%) treatments than in the C group (5.27%), whereas the sum of the humic‐like acid fraction carbon (HAC) and fulvic‐like acid fraction carbon (FAC) was in the order of Inoculation (10.31%) > AC (6.87%) > C (1.91%). It is well‐known that the humification degree of humic‐like and fulvic‐like acids is markedly higher than that of the hydrophilic (HyI) fraction. These results indicated that the humification degree was markedly higher in the Inoculation and AC treatments than in the C group. However, compared with the Inoculation group, it was lower in the AC treatment. Wei *et al*. (2014) reported that compost with a low degree of humification was beneficial to the remediation of polluted soils, whereas compost with a high degree of humification was conducive to preserving the moisture and fertility of soil. The inoculation compost in this study might be conducive to preserving the moisture and fertility of soil.

### Fluorescence spectra

#### Excitation–emission matrix (EEM) spectra

**Figure 3:**
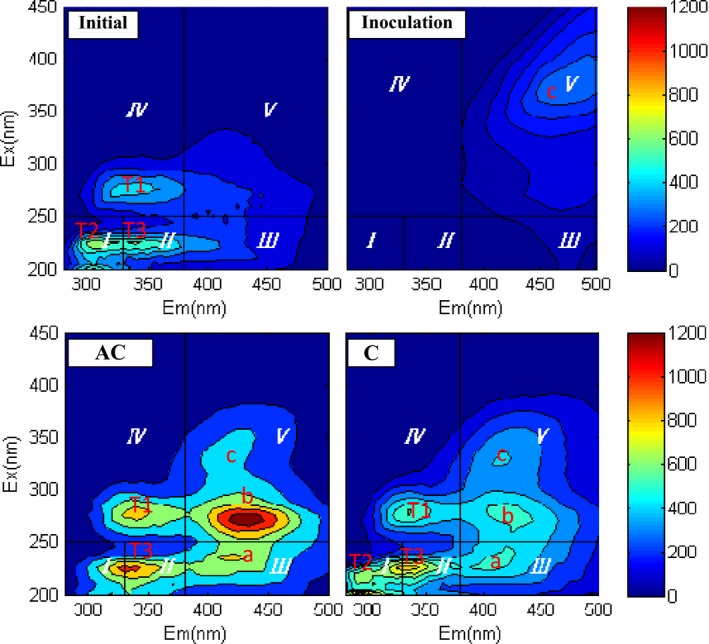
Excitation–emission matrix spectra of DOM extracted from the raw material (Initial) and compost products from the different composting treatments. Inoculation – the inoculation with MCDOA, AC – the alkaline compound treatment, C – the control group.

The three‐dimensional EEM fluorescence spectra of the dissolved organic matter (DOM) samples at the start and end of the three composting treatments are illustrated in Fig. 3. The EEM contour of the DOM from the initial samples was clearly different from those of the samples at the end of composting. The former mainly exhibited three peaks: T1, T2 and T3. According to Marhuenda‐Egea *et al*. (2007), peak T1 is associated with soluble microbial byproduct‐like substances and the peaks T2 and T3 are involved in aromatic protein‐like fluorescence, such as tyrosine and tryptophan. In addition to the aforementioned three peaks, the EEM spectra of DOM extracted from the AC and C samples were also characterized by the peaks a, b and c. According to He *et al*. (2011b), peak a is representative of fulvic acid‐like material and peaks b and c are associated with humic acid‐like fluorophores. Compared with the AC and C samples, peaks T1, T2, T3, a and b disappeared in the EEM spectra of DOM extracted from the Inoculation compost, whereas the humic‐like peak c became the primary fluorescence peak. Furthermore, the excitation/emission wavelength pairs (EEWPs) of peak c shifted markedly towards longer wavelengths (red shift). This red shift may be attributed to an increase in aromaticity and polycondensation structures of the humic‐like organic substances (Wei *et al*., 2007). The results indicated an increase in the molecular size, aromatic polycondensation, and a larger degree of humification in the Inoculation compost (Senesi *et al*., 1991). Compared with the C compost, peak T2 disappeared in AC, which suggested that the addition of alkaline compounds could promote the degradation of tyrosine. In addition, the humic‐ and fulvic‐like peaks (a, b and c) exhibited an obvious increase in fluorescence intensity in AC. The biostabilization of composting organic matter includes several stages: the disappearance of some easily biodegradable compounds like protein‐like materials, the formation of humic‐ and fulvic‐like substances with a low degree of aromatic polycondensation and the synthesis of humic macromolecules by the transformation of humic‐ and fulvic‐like materials with simple structures (He *et al*., 2011a). Generally, fulvic‐like compounds and humic‐like substances with short wavelengths first increased and then decreased with composting time. Thus, compared with the C compost, the increase in peaks a, b, c might indicate that addition of alkaline compounds could accelerate the humification process in food waste composting to a certain extent. Peaks T1 and T3 also presented a significant increase in fluorescence intensity, which may be due to the higher microbial activity in the AC compost than in the C group. It should be noted that compared with the initial sample, fluorophores T1, T2 and T3 showed an increase in peak intensity in the AC and C samples, which might result from microbial metabolism.

#### Fluorescence regional integration (FRI) analysis of DOM

**Figure 4:**
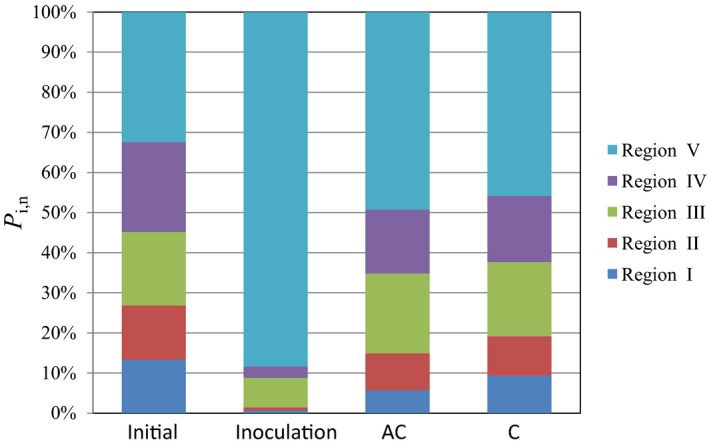
The volumetric fluorescence distribution of DOM extracted from the raw material (Initial) and compost products from different composting treatments. Inoculation – the inoculation with MCDOA, AC – the alkaline compound treatment, C – the control group.

Fluorescence regional integration has been used as an analytical tool for quantifying the EEM spectra of DOM from compost (Wei *et al*., 2016b). According to Chen *et al*. (2003), the EEMs of DOM were divided into five regions (Fig. 4). Regions I, II, III, IV and V are for tyrosine, tryptophan and fulvic acid‐like, soluble microbial byproduct‐like and humic‐like materials respectively. In general, the percent fluorescence response (*P*_i,n_) value of DOM in mature compost can be characterized as decreasing in Regions I and II (simple aromatic proteins) and increasing in Regions III and V (fulvic‐like and humic‐like materials) compared with the raw material. The distribution of *P*_i,n_ values in these five regions in the raw material was more well‐proportioned. The *P*_i,n_ values in Regions I, II, III, IV and V were 13.25%, 13.62%, 18.26%, 22.45% and 32.43% respectively. After composting, the distribution of *P*_i,n_ values changed significantly in the inoculation compost. The *P*_i,n_ values in Regions I, II and IV decreased by 95.11%, 94.19% and 87.41%, respectively, which demonstrated that the simple aromatic proteins and soluble microbial byproduct‐like materials decreased after composting. In contrast, the *P*_i,n_ in Region V increased by 172.57%, which revealed an increase in large molecular humic‐like materials. Compared with the raw material, the AC and C compost also showed a decrease in *P*_i,n_ values in Regions I, II and IV and an increase in the *P*_i,n_ in Region V. However, the decreased and increased levels were markedly higher in the inoculation compost than in the AC and C groups. These findings indicated that inoculation with MCDOA could promote the degradation of simple compounds, such as proteinaceous materials, and the formation of complicated large molecular structures. It should be noted that compared with the raw material, the *P*_i,n_ in Region III decreased by 59.76% in the Inoculation treatment and increased by 8.98% and 1.26% in the AC and C groups respectively. Region III stands for fulvic acid‐like materials, which have a lower molecular weight and degree of aromatic polycondensation compared with humic‐like materials. Generally, the humic acid‐like materials originated from and were transformed by fulvic acid‐like substances. Therefore, the aforementioned phenomenon revealed that the composting efficiency was markedly higher in the Inoculation group than in the AC and C treatments. In addition, compared with the C treatment, AC presented higher *P*_i,n_ values in Regions I, II and IV and lower *P*_i,n_ values in Regions III and V. This indicated that to a certain extent, the addition of alkaline compounds could also enhance the composting efficiency and improve the humification degree of compost.

### Bacterial community dynamics and composition

#### Bacterial community dynamics

**Figure 5:**
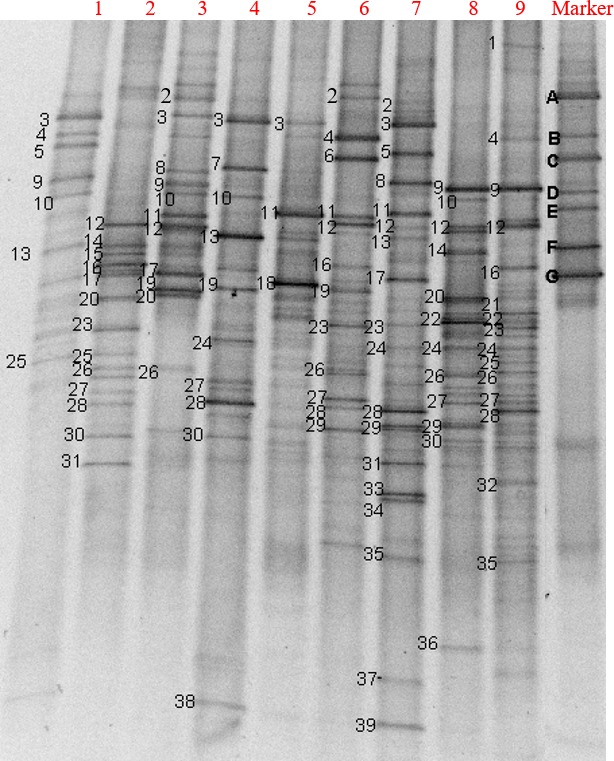
DGGE profile of 16S rRNA gene fragments amplified with the primers R534 and GC‐F341. Band patterns of three composting treatments are presented. The numbers above each lane indicate the sampling stages. 1–C (the mesophilic stage);2–C (the thermophilic stage);3–C (the mature stage);4–AC (the mesophilic stage);5–AC (the thermophilic stage);6–AC (the mature stage);7–Inoculation (the mesophilic stage);8–Inoculation (the thermophilic stage);9–Inoculation (the mature stage);Marker–microbial consortium degrading organic acids. Inoculation – the inoculation with MCDOA, AC – the alkaline compound treatment, C – the control group.

The DGGE band patterns of 16S rDNA fragments are shown in Fig. 5. The closest relatives of bands excised from the DGGE profile are shown in Table 1. PCR‐DGGE profiles of the bacterial communities implied that the bacterial dynamics and distribution for the Inoculation treatment visibly differed from those for the AC and C groups. The quantity of bands in the Inoculation lanes clearly exceeded that in the AC and C lanes. The Inoculation treatment exhibited a markedly higher bacterial diversity (Fig. S2). These results revealed that inoculation with MCDOA could change the bacterial community structure and improve bacterial diversity in the course of food waste composting. In addition, it could be observed that several bands emerged only in the DGGE profiles from the Inoculation treatment, such as bands 1, 5, 21, 22, 32, 33, 34, 35, 36, 37 and 39. However, these bands did not derive from the MCDOA inoculum. This finding indicated that inoculating with MCDOA could stimulate the generation of new bacterial species, especially in the mesophilic stage of food waste composting. Bands A, B, C, D, E, F and G were the dominant bacterial species of MCDOA. It could be found that these bands also emerged in the DGGE profiles from the Inoculation treatment by comparing Table 1 and the dominant bacterial species of MCDOA (shown in the second subsection under ‘Experimental procedures’). This phenomenon suggested that the MCDOA inoculum, which originated from a food waste compost sample, was well adapted to the food waste composting conditions and played a part in degrading low molecular weight organic acids in food waste. In comparison with the C group, the AC treatment displayed a lower quantity of DGGE bands at the mesophilic and thermophilic stages and a larger quantity of DGGE bands at the mature stage (Fig. S2). At the same time, the DGGE band distribution also showed obvious changes. These findings revealed that to a certain extent, the addition of alkaline compounds could decrease bacterial diversity and change the bacterial community composition. However, as bacteria gradually adapted to the environmental changes in compost due to the addition of alkaline compounds, the bacterial diversity recovered at the mature stage.

**Table 1:** Nucleotide sequences of the bacteria related to the DGGE bands shown in Fig. 5

Band name	Accession no.	Closest relatives	Similarity (%)
1	AY911716.1	Uncultured *Legionella* sp.	99
2	KJ703076.1	Uncultured *Dysgonomonas* sp.	100
3	KJ914897.1	*Weissella confusa* strain	100
4	KC634287.1	*Pseudomonas caeni* strain	100
5	JX467643.1	*Aeribacillus pallidus* strain	100
6	KJ726611.1	*Pseudomonas* sp.	100
7	JN173175.1	Uncultured *Streptococcus* sp.	100
8	KF994925.1	*Lactobacillus coryniformis* strain	97
9	JQ246806.2	*Pseudomonas* sp.	100
10	KC133394.1	Uncultured *Lactobacillus* sp.	99
11	KF303794.1	*Lactobacillus salivarius* strain	100
12	HQ912777.1	Uncultured compost bacterium	100
13	KJ803948.1	*Streptococcus equinus* strain	100
14	KC178607.1	*Bacillus thuringiensis* strain	100
15	KF026352.1	*Sporosarcina globispora* strain	99
16	HG937599.1	*Bacillus* sp.	100
17	KF527207.1	*Bacillus cereus* strain	100
18	AY211115.1	*Bacillus* sp.	100
19	AP007209.1	*Bacillus cereus* strain	98
20	AP008934.1	*Staphylococcus saprophyticus* strain	100
21	JN866379.1	Uncultured *Deinococcus* sp.	100
22	HE802007.1	Uncultured bacterium	99
23	KF202776.1	*Pantoea* sp.	100
24	KF436776.1	*Pantoea agglomerans* strain	100
25	KC935383.1	*Serratia grimesii* strain	100
26	GU227536.1	*Pseudomonas aeruginosa* strain	98
27	KJ730230.1	Uncultured bacterium	100
28	KJ722533.1	*Bacillus smithii* strain	100
29	KC493201.1	*Bacillus* sp.	100
30	HM469546.1	Uncultured bacterium	99
31	KF527200.1	*Bacillus thuringiensis* strain	99
32	KJ190161.1	*Aneurinibacillus thermoaerophilus* strain	100
33	AF489284.1	*Sphingobacterium* sp.	100
34	KC148580.1	*Escherichia coli* strain	98
35	EF122436	*Sphingobacterium composti* strain	95
36	AJ244699	*Flavobacterium* sp.	96
37	EF633293.1	*Bacillus mycoides* strain	100
38	JQ775349.1	Uncultured *Thermobacillus* sp.	100
39	KM047433.1	Uncultured *Pusillimonas* sp.	100

#### Analysis of 16S rDNA sequences

The sequences of a total of 39 DNA fragments were successfully determined (shown in Table 1). Table S2 showed 18 microbial species that were capable of secreting key enzymes for the acetate and propanoate metabolic pathways and were detected by proteomic technology in our previous study (Song *et al*., 2018). There are 13 species belonging to *Bacteria*. It was observed that bands 1, 21, 34 and 37 of the 11 typical bands of the Inoculation treatment were capable of secreting key enzymes for the acetate and propanoate metabolic pathways by comparing Tables 1 and S2. They belonged to *Legionella* sp., *Deinococcus* sp., *Escherichia* sp. and *Bacillus* sp. respectively. This phenomenon suggested that the inoculation with MCDOA could stimulate the generation of bacterial species capable of secreting key enzymes for the acetate and propanoate metabolic pathways. It was at the mesophilic stage of the Inoculation treatment that bands 34 and 37 were detected (at this time, microbial activity was inhibited by the acidification of composting material in the C group). This finding might account for why inoculation with MCDOA could rapidly degrade low molecular weight organic acids, and thus avoid the lag phase of the pile temperature rise in the Inoculation treatment. Karadag *et al*. (2013) reported that *Sphingobacterium* and *Flavobacterium* could degrade lignin. The degradation of lignin during composting is closely related to the humification process of composting substrate (Song *et al*., 2016). In the current study, bands 33 (*Sphingobacterium*) and 35 (*Sphingobacterium*), band 36 (*Flavobacterium*) and band 35 emerged at the mesophilic, thermophilic and mature stages of the inoculation group respectively. This phenomenon indicated that inoculation with MCDOA stimulated the generation of bacterial species that were capable of degrading lignin throughout the entire course of food waste composting. This might account for the high degree of humification of the Inoculation compost (sections ‘Humic‐like extract carbon (HEC)’ and ‘The composition of carbon fractions’).

In addition to bands 1, 21, 34 and 37, bacteria that were related to acetate and propanoate metabolism also included *Bacillus* (bands 14, 16, 17, 18, 19, 28, 29 and 31), *Pseudomonas* (bands 4, 6, 9 and 26), *Staphylococcus* (band 20) and *Streptococcus* (bands 7 and 13) (shown in Table S2). Because microbial activity was inhibited by acidification of the composting material primarily occurring in the mesophilic stage, bacterial species that were related to acetate and propanoate metabolism were compared only in the mesophilic stage of the three composting treatments in this study. In the mesophilic stage, four bands were detected in the C (bands 4, 6, 9 and 13) and AC (bands 7, 13, 19 and 28) groups. Seven bands (bands 13, 17, 28, 29, 31, 34 and 37) were detected in the Inoculation group. These findings indicated that inoculating with MCDOA improved the diversity of bacteria that were capable of degrading acetic and propionic acids. Compared with the C group, the diversity of bacteria that could degrade acetic and propionic acids did not change significantly in the mesophilic stage of the AC group, but the bacterial species changed. It could be inferred that the MCDOA inoculum enhanced the degradation rate of low molecular weight organic acids primarily by improving the diversities of acetic and propionic acid degrading bacteria, whereas the mechanism of avoiding a disruption of the increase in pile temperature in the AC group may be that the alkaline amendment was effective at providing a good environment for microbial degradation of organic acids, just as exhibited in the study of Chan *et al*. (2016).

In the current study, *Aeribacillus* (band 5), *Aneurinibacillus* (band 32), *Dysgonomonas* (band 2), *Lactobacillus* (bands 8, 10 and 11), *Pantoea* (bands 23 and 24), *Pusillimonas* (band 39), *Serratia* (band 25), *Sporosarcina* (band 15), *Thermobacillus* (band 38), *Weissella* (band 3) and an uncultured bacterium (bands 12, 22, 27 and 30) were also detected. Wakase *et al*. (2008) reported that *Lactobacillus* was detected in many composting processes, was dominant in the early stages of composting and was capable of producing acetic acid. In this study, *Lactobacillus* was detected in the mesophilic stage of the three composting treatments, but it should be noted that three *Lactobacillus* species were still detected in the mature stage of the C group. Furthermore, *Lactobacillus* bacteria were observed during the entire composting process of the AC treatment. In contrast, *Lactobacillus* was not detected in the mature period of the Inoculation group. The high diversity of *Lactobacillus* bacteria in the mature stage of the C group may be related to the lower pH value. Touzel *et al*. (2000) reported that *Thermobacillus* sp. was a gram‐negative, spore‐forming, aerobic, non‐motile, rod‐shaped thermophilic bacterium. *Thermobacillus xylanilyticus* was a representative species of *Thermobacillus* and capable of degrading xylan. Rastogi *et al*. (2010) reported that *Thermobacillus* sp. were thermophilic bacteria and could degrade plant cell wall material (the predominant hemicellulose component of a plant cell wall is xylan). In this study, *Thermobacillus* was detected only in the mesophilic stage of the AC treatment. Francis *et al*. (2000) reported that *Pantoea agglomerans* (band 24) are facultative anaerobes and capable of transferring electrons using acetic acid as the electron donor and Fe (III) as an electron acceptor and simultaneously degrading complex organic matter. In this study, *Pantoea agglomerans* was detected during the entire composting process of the Inoculation treatment, which indicated that *Pantoea agglomerans* played an important role in acetic acid metabolism and thus in overcoming the acidification problem of composting the material in the Inoculation group. *Pantoea agglomerans* was also detected in the mesophilic stage of the AC group, which indicated that *Pantoea agglomerans* might also play a part in overcoming the acidification problem in the initial stage of the AC group. *Weissella* was able to produce lactic acid, some of which is often used in food production and preservation processes, such as traditional kimchi (Choi *et al*., 2002). In the C group, *Weissella* was detected in the mesophilic and mature periods. In the AC group, *Weissella* was detected in the mesophilic and thermophilic period. However, in the Inoculation treatment, Weissella was detected only in the mesophilic period. *Aneurinibacillus* sp. is a thermophilic acidophilic bacterium and could utilize lactic acid (Allan *et al*., 2005). *Aneurinibacillus* was detected only in the mature stage of the Inoculation group. These findings showed that compared with the AC and C groups, the Inoculation treatment displayed fewer bacterial species capable of producing lactic acid and more bacterial species capable of degrading acetic acid, which may be related to the absence of accumulation of organic acids and the pH drop in the Inoculation treatment.

## Conclusions

Microbial consortium for the degradation of organic acids, which acts synergistically in the degradation of organic acids, was inoculated in food waste compost. Inoculation with MCDOA could avoid the lag phase in the pile temperature rise due to acidification of the composting material, enhance composting efficiency and effectively shorten the composting period. Moreover, it also could promote degradation of simple proteinaceous compounds and formation of complicated humic‐like substances. The PCR‐DGGE results indicated that inoculation with MCDOA could stimulate the generation of bacterial species capable of secreting key enzymes for the acetate and propanoate metabolic pathways and degrading the lignin, which might account for the high composting efficiency and degree of humification of the Inoculation group.

## Experimental procedures

### Composting materials

The food waste was obtained from the dining hall of the Chinese Research Academy of Environmental Science (Beijing, China). The refractory matter such as napkins and bone were manually separated and discarded. Residual samples were minced into pieces with a diameter of less than 5 mm by a food waste pulverizer and mixed well before the composting experiments began. Wheat bran was added as a bulking agent to adjust the ratio of carbon/nitrogen (C/N) and moisture of the composting substrate. It was acquired from the Chinese Research Academy of Environmental Science. The chemical characteristics of the food waste were as follows: a C/N ratio of 23.3, an organic matter concentration of 79.9% and a moisture concentration of 78.7%. Some basic characteristics of the wheat bran were a C/N ratio of 32.4, an organic matter concentration of 89.3% and moisture concentration of 10.2%.

### Source, construction and composition of MCDOA

The microbial consortium originated from the initial phase of food waste composting when the pH of the composting material maintained a range of 4–5 for 1 month. A 5‐g sample was used as the source of the microorganisms, was transferred into 50 ml of sterile water, and was incubated at room temperature for 1 h in an orbital shaker incubator with a revolving speed of 150 rpm. After standing, 1 ml of the suspension was added to each of 12 autoclaved flasks (each containing 50 ml of Luria–Bertani medium (Sigma™, St. Louis, Mo, USA)). In order to simulate the pH conditions of the initial food waste composting, the pH of the Luria–Bertani medium was adjusted to 5.3 by mixed acids, which consisted of acetic, propionic, butyric and lactic acids. In the acid mixture, the mass ratio of the above four acids were 3:3:2.5:12.5. The mass ratio was set according to an acidified food waste compost microenvironment (Nakasaki *et al*., 2013)) and incubated at 50°C for 24 h in an orbital shaker incubator with a revolving speed of 200 rpm. The cultures were subcultured according to the above procedure. In the course of continuous subcultures, the dynamic changes in pH in the medium were monitored by pH paper, and the amount of the mixed acid in the medium was stepwise increased. After culturing for 50 generations in succession, the culture that exhibited the highest degradation rate of low molecular weight organic acids was selected. DGGE profiles also showed that the community structure of the selected culture was stable (shown in supplementary Fig. S3). A high‐efficiency MCDOA was obtained (the pH value of the medium increased from 5.30 to 7.59 after cultivating the MCDOA for 24 h).

The microbial consortium is composed of bacteria, and the cooperation and symbiosis of the contained microbes enhance the degradation of organic acids. The MCDOA was composed of *Dysgonomonas* sp., *Pseudomonas caeni* strain, *Aeribacillus pallidus* strain, *Pseudomonas* sp., *Lactobacillus salivarius* strain, *Bacillus thuringiensis* strain and *Bacillus cereus* strain.

### Composting operation

The composting process was carried out in a reactor, the core part of which was a cylinder with a diameter of 330 mm and a height of 400 mm. The volume of the cylinder was approximately 34 l. A 6‐mm trachea was connected to the top of the reactor, and a perforated metal plate was used at the bottom to hold the composting substrates and to distribute air equally. The composting system was connected to a blower that provided the reactor with air from the bottom. The wall of the cylinder was equipped with a detector for recording the temperature of the composting substrates.

The food waste and wheat bran were manually mixed thoroughly (*w* (food waste): *w* (wheat bran) = 8:2, on a dry basis). The basic characteristics of the composting mix were a C/N ratio of 25.08 and a moisture concentration of 55.7%. Up to 34 l of the composting mixture was used in each composting experiment. Three composting experiments were conducted. In the inoculation group (Inoculation), the aforementioned microbial consortium was added to the composting mix at the level of 1.25 ml kg^−1^ dry composting mix (the inoculation quantity was set according to our previous studies (Song *et al*., 2016)), and the concentrations of the strains were about 1 × 10^8^ CFU ml^−1^. In the alkaline compound treatment group (AC), magnesium oxide (MgO) and dipotassium hydrogen phosphate (K_2_HPO_4_) were added to alleviate the drop in pH due to the intensive acidification at the initial stage of food waste composting. The dosages of MgO and K_2_HPO_4_ were 0.05 and 0.1 M kg^−1^ dry composting mix, respectively (the dosages were set according to Wang *et al*. (2013)). Nothing was added in the control group (C). In the course of composting, the ventilation rate was set at 0.5 l min^−1^ kg^−1^ composting mix (in dry solid weight). The temperature was measured every 12 h. The composting mixtures were manually turned, and their moisture was adjusted to 50–60% again.

Before each sampling, the composting mass in the reactors was mixed thoroughly with a shovel. Samples were subsequently withdrawn from different levels of the central reactors on days 0, 3, 8, 13, 19, 35 and 47. After mixing, subsamples of approximately 100 g were stored at a temperature of −20°C before further analysis. The measurements of physico‐chemical parameters, the germination index (GI) and spectra were performed 3 times per sample.

### Physicochemical analysis

The C/N ratio of the FW and wheat bran (previously freeze‐dried and crushed) was determined using an Elementar Vario El elemental analyzer. Determination of the moisture content of the fresh samples was conducted by the loss of weight after drying the samples at 105°C for 24 h in a drying oven, while the organic matter content of the solid samples was determined by heating the samples at 550°C for 6 h in a PYRO 260 muffle furnace (MILESTONE, Italy). The temperatures of the compost piles were monitored with a Thermo Recorder RTW‐30S (Espec, Osaka, Japan). The aqueous extracts (solid to water ratio of 1:10, w/v) of the composting samples were prepared for measuring the pH, electrical conductivity and germination index. The measurement of pH and EC were conducted using a Mettler Toledo S20K pH meter (Mettler Toledo Instruments, Shanghai, China) and an EC meter (YSI, Model 30M, 100FT, USA), respectively. The GI was determined according to Hosseini and Aziz (2013). Extraction and fractionation of humic substances were conducted according to our previous study (Song *et al*., 2016). The total organic carbon (TOC) of freeze‐dried composting samples, as well as HEC, HyIC, HAC and FAC were determined using a multi N/C 2100 TOC/TN analyzer (Analytikjena, Jena, Germany).

### Fluorescence spectroscopy of DOM

The DOM was obtained according to He *et al*. (2011a). Briefly, the composting samples were extracted with deionized water using a sample: extractant ratio of 1:10 (w/v) for 24 h in a horizontal shaker (a rotational speed of 200 rpm) at 20°C. The obtained supernatant was then centrifuged at 10,000 rpm for 10 min, filtered through a 0.45‐μm membrane filter, and freeze‐dried.

The EEM fluorescence spectra of the DOM were obtained at a scan speed of 2400 nm min^−1^ over the 200–450 nm and 280–520 nm ranges of excitation and emission wavelengths, respectively, and the excitation wavelength increment was set at 5 nm. After regulating the scattering using interpolation in the areas affected by first‐ and second‐order Rayleigh and Raman scatter, the FRI technique was adopted for analysis (Chen *et al*., 2003).

### Analysis of bacterial community

The diversity and changes in the bacterial community of the compost were determined by the PCR–DGGE technique. The extraction and purification of DNA, preparation of the PCR reaction mixture and operation of PCR program, and DGGE analyses were carried out according to previous work (Song *et al*., 2016).

The dominant bands were excised and sequenced according to Nakasaki *et al*. (2013). The results are shown in Table 1.

### Statistical analysis

The physico‐chemical analysis and GI data were subjected to analysis of variance (ANOVA) using spss version 17.0 (SPSS Inc., Chicago, IL, USA). A value of *P *< 0.05 was considered statistically significant. The digital image of the DGGE profile was analysed by quantity one v4.62 (Bio‐Rad, Hercules, CA, USA) software.

## Conflict of interest

None declared.

## Supporting information


**Fig. S1.** The carbon nitrogen ratio of raw material and three compost.
**Fig. S2.** Changes of Shannon–Winner index and the quantity of DGGE bands of bacteria during different composting treatments.
**Fig. S3.** DGGE profiles of MCDOA from the 46th to 50th generation after 24 h‐cultivation in culture medium.
**Table S1.** Changes in concentration of short chain organic acids during different composting treatments.
**Table S2.** The strains secreting key enzymes of acetate and propanoate metabolic pathways.
