# Supplementary material for: Effect of inoculation with a microbial consortium that degrades organic acids on the composting efficiency of food waste
Source: Microb Biotechnol. 2018 Jul 2;11(6):1124–36. doi: 10.1111/1751-7915.13294 (PMC6196389; doi:10.1111/1751-7915.13294)
Supplement: Supplementary file 1 — Fig. S1. The carbon nitrogen ratio of raw material and three compost. Fig. S2. Changes of Shannon–Winner index and the quantity of DGGE bands of bacteria during different composting treatments. Fig. S3. DGGE profiles of MCDOA from the 46th to 50th generation after 24 h‐cultivation in culture medium. Table S1. Changes in concentration of short chain organic acids during different composting treatments. Table S2. The strains secreting key enzymes of acetate and propanoate metabolic pathways. [file MBT2-11-1124-s001.doc]

**Supplementary Material**


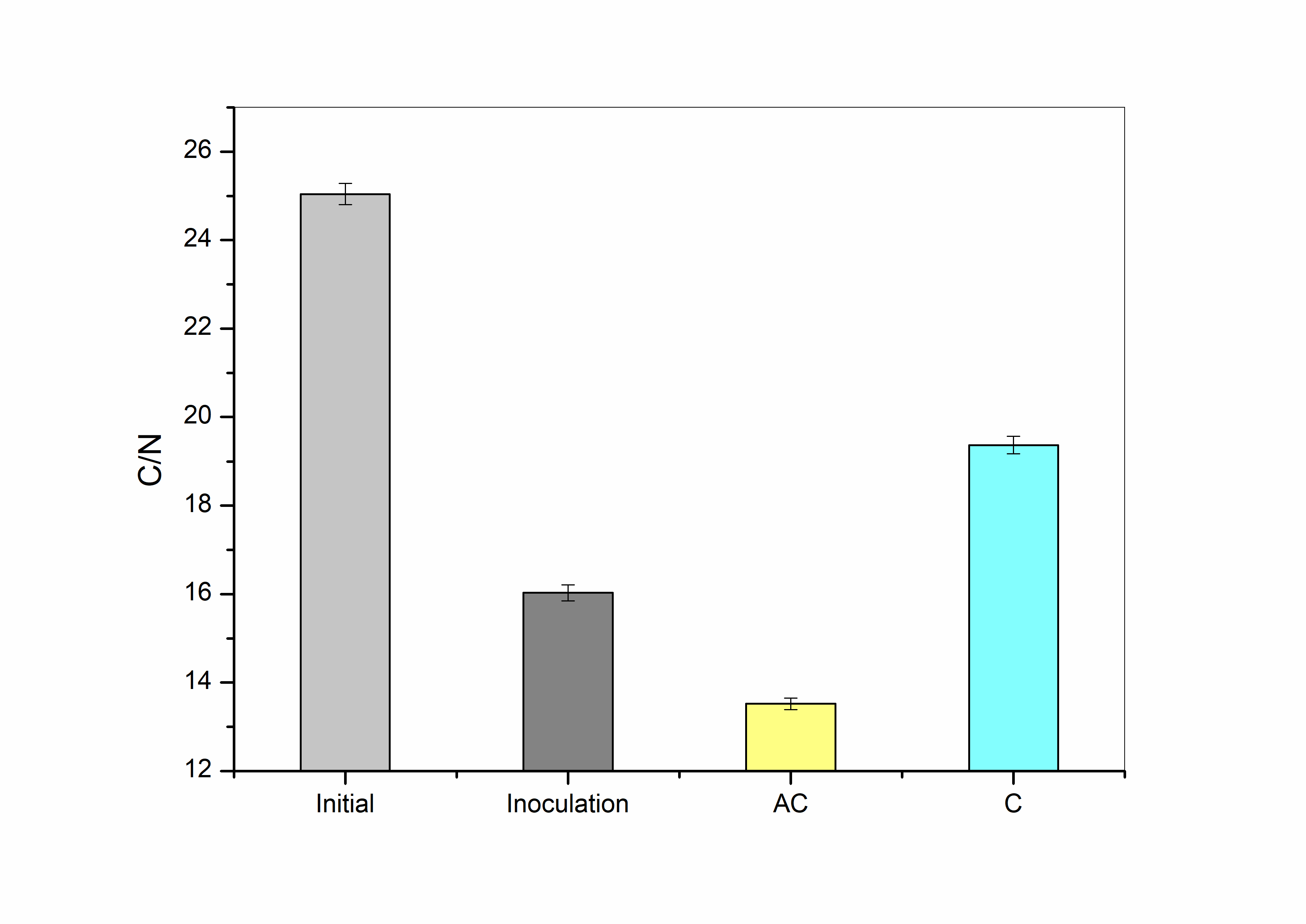


Fig. S1. The carbon nitrogen ratio of raw material and three compost. Inoculation–the inoculation with MCDOA, AC–the alkaline compound treatment, C–the control group. Data represent the mean of three replicates and the error bars are standard deviation.


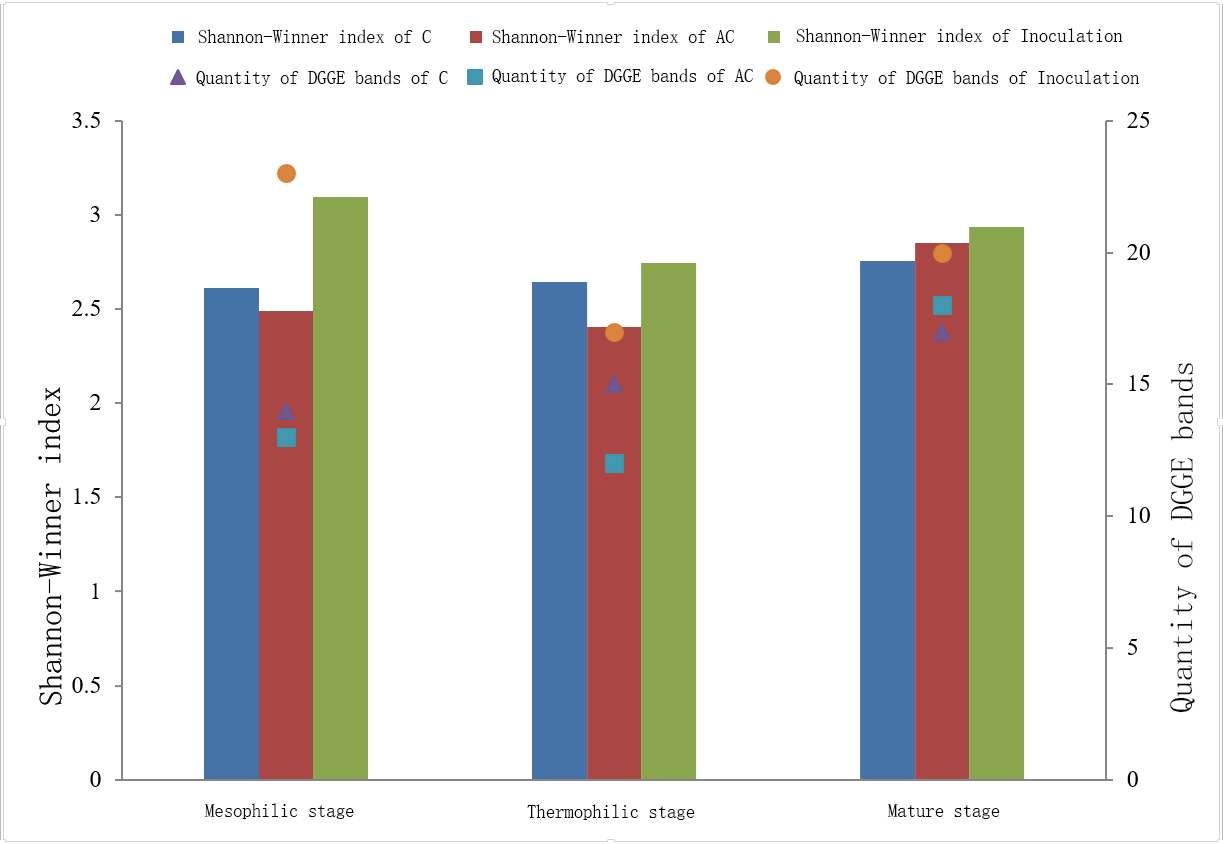


Fig. S2. Changes of Shannon-Winner index and the quantity of DGGE bands of bacteria during different composting treatments.


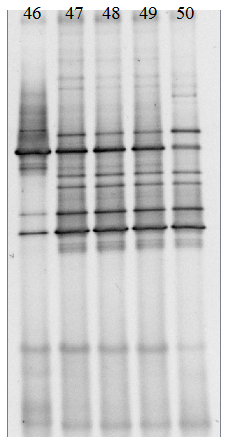


Fig. S3. DGGE profiles of MCDOA from the 46^th^ to 50^th^ generation after 24h-cultivation in culture medium.

Table S1 Changes in concentration of short chain organic acids during different composting treatments. Inoculation–the inoculation with MCDOA, AC–the alkaline compound treatment, C–the control group.

| Sampling time  (d) | Acetic acid  (mg/L) | Propanoic acid (mg/L) | Butyric acid  (mg/L) | Lactic acid  (mg/L) |
| --- | --- | --- | --- | --- |
| Inoculation |  |  |  |  |
| 0 | 1698.91 (24.12)^k*^ | 1405.69 (23.75)^n^ | 1400.34 (28.14)^p^ | 1754.23 (30.47)^p^ |
| 3 | 1002.33 (16.35)^h^ | 1002.37 (15.28)^k^ | 975.35 (14.25)^l^ | 1433.68 (27.44)^m^ |
| 8 | 783.21 (9.42)^f^ | 678.99 (8.96)^hi^ | 536.67 (8.27)^g^ | 1085.97 (14.25)^k^ |
| 13 | 342.19 (5.39)^d^ | 521.33 (6.24)^f^ | 423.93 (5.49)^f^ | 734.25 (9.22)^g^ |
| 19 | 319.23 (5.27)^d^ | 532.87 (6.00)^f^ | 362.39 (4.91)^d^ | 737.44 (9.03)^g^ |
| 35 | 110.57 (4.01)^a^ | 356.23 (5.19)^c^ | 349.73 (4.65)^d^ | 483.87 (5.64)^d^ |
| 47 | 84.33 (2.98)^a^ | 179.00 (3.66)^a^ | 73.57 (2.60)^a^ | 256.39 (4.38)^a^ |
| AC |  |  |  |  |
| 0 | 1709.23 (29.88)^k^ | 1417.37 (22.84)^n^ | 1389.46 (20.45)^p^ | 1738.24 (24.33)^p^ |
| 3 | 1274.87 (18.20)^i^ | 1233.64 (18.07)^l^ | 1068.36 (14.97)^m^ | 1567.70 (26.24)^n^ |
| 8 | 953.93 (14.13)^g^ | 993.74 (13.99)^k^ | 746.44 (9.55)^j^ | 1259.66 (18.35)^l^ |
| 13 | 445.37 (5.29)^e^ | 688.29 (8.88)^i^ | 563.47 (7.64)^h^ | 839.48 (10.02)^h^ |
| 19 | 440.21 (5.16)^e^ | 532.11 (6.39)^f^ | 631.54 (7.90)^i^ | 683.50 (7.96)^f^ |
| 35 | 259.30 (3.39)^c^ | 487.38 (5.81)^e^ | 387.91 (5.04)^e^ | 555.38 (6.59)^e^ |
| 47 | 174.39 (3.87)^b^ | 233.58 (4.09)^b^ | 198.46 (3.96)^b^ | 313.68 (4.57)^b^ |
| C |  |  |  |  |
| 0 | 1708.82 (28.46)^k^ | 1370.44 (20.83)^m^ | 1405.34 (21.05)^p^ | 1702.88 (27.97)^o^ |
| 3 | 1840.07 (30.81)^m^ | 1482.58 (22.58)^o^ | 1283.91 (19.20)^n^ | 2200.55 (35.12)^r^ |
| 8 | 1787.25 (29.98)^l^ | 1699.93 (29.31)^p^ | 1347.00 (19.99)^o^ | 1808.68 (31.09)^q^ |
| 13 | 1394.85 (24.75)^j^ | 865.99 (13.27)^j^ | 995.03 (11.27)^l^ | 1012.76 (16.08)^j^ |
| 19 | 759.85 (10.07)^f^ | 656.27 (7.82)^h^ | 788.24 (10.43)^k^ | 879.54 (14.15)^i^ |
| 35 | 778.38 (10.11)^f^ | 598.76 (6.84)^g^ | 556.32 (7.88)^gh^ | 664.38 (8.04)^f^ |
| 47 | 467.53 (5.92)^e^ | 400.38 (5.36)^d^ | 267.89 (4.00)^c^ | 434.85 (5.39)^c^ |

*Values followed by different letters (a-r) are statistically significantly different (p < 0.05). Values in parenthesis are standard deviations.

Table S2 The strains secreting key enzymes of acetate and propanoate metabolic pathways.

| No. | Generic name | Class name |
| --- | --- | --- |
| 1 | *Staphylococcus* | *Bacteria* |
| 2 | *Pseudomonas* | *Bacteria* |
| 3 | *Bacillus* | *Bacteria* |
| 4 | *Escherichia* | *Bacteria* |
| 5 | *Buchnera* | *Bacteria* |
| 6 | *Yersinia* | *Bacteria* |
| 7 | *Bradyrhizobium* | *Bacteria* |
| 8 | *Geobacillus* | *Bacteria* |
| 9 | *Rhodopseudomonas*（细菌） | *Bacteria* |
| 10 | *Legionella* | *Bacteria* |
| 11 | *Deinococcus* | *Bacteria* |
| 12 | *Streptococcus* | *Bacteria* |
| 13 | *Rickettsia* | *Bacteria* |
| 14 | *Corynebacterium* | *Actinobacteria* |
| 15 | *Mycobacterium* | *Actinobacteria* |
| 16 | *Rhodococcus* | *Actinobacteria* |
| 17 | *Saccharomyces* | *Fungi* |
| 18 | *Candida* | *Fungi* |
